# Key determinants of self-management in patients with non-dialysis-dependent chronic kidney disease: a systematic review

**DOI:** 10.3389/phrs.2026.1609108

**Published:** 2026-06-30

**Authors:** Alimzhan Muxunov, Dina Kalinina, Ikechi Okpechi, Racquel Lowe-Jones, Abduzhappar Gaipov, Zhanat Kuanshaliyeva, Symbat Bayakhmetova, Meruyert Madikenova, Antonio Sarria-Santamera

**Affiliations:** 1 Department of Biomedical Sciences, Nazarbayev University School of Medicine, Astana, Kazakhstan; 2 Division of Nephrology, University of Alberta, Edmonton, AB, Canada; 3 Department of Internal Medicine, Cayman Islands Health Services Authority, George Town, Cayman Islands; 4 Clinical Academic Department of Internal Medicine, CF “University Medical Center”, Astana, Kazakhstan

**Keywords:** chronic kidney disease, health literacy, knowledge, self-care, self-efficacy

## Abstract

**Objectives:**

Adoption of recommended self-management behaviors has been associated with a lower risk of disease progression, cardiovascular morbidity, and mortality in patients with chronic kidney disease (CKD). This systematic review aims to identify factors associated with the adherence of non-dialysis-dependent CKD (NDD-CKD) patients to recommended self-management practices.

**Methods:**

Following PRISMA guidelines, we searched PubMed, Embase, Scopus, Medline, and CINAHL Plus databases from 1st January 2010 to 30th June 2024. The extracted data included associations of variables with self-management scores. The findings were descriptively presented in a grouped tabular form. The study was registered with the PROSPERO, CRD42024547304.

**Results:**

From 1,914 studies identified, 16 cross-sectional studies with 3,658 participants were selected for inclusion. Objective CKD knowledge, health literacy, self-efficacy and social support consistently showed positive associations with self-management across multiple studies.

**Conclusion:**

This systematic review revealed multiple factors associated with self-management in NDD-CKD patients, with disease-related knowledge, health literacy, and self-efficacy showing the most consistent positive associations across studies. Healthcare providers and policymakers should develop and implement health literacy and awareness programs as a strategy for improving patient outcomes.

**Systematic Review Registration:**

https://www.crd.york.ac.uk/prospero/display_record.php?RecordID=547304, identifier CRD42024547304.

## Introduction

Chronic kidney disease (CKD) is a growing global health concern, affecting more than 10% of the global population [1]. CKD significantly impairs individuals’ physical health and quality of life, increasing the risk of cardiovascular disease (CVD), disability, and premature mortality. In 2017, CKD accounted for 1.2 million deaths and loss of 35.8 million disability-adjusted life years (DALYs) [2].

CKD is characterized by progressive irreversible loss of kidney function in five stages (G1-G5). Each subsequent stage of CKD is associated with a higher incidence of complications, mortality, and treatment costs [3]. In the final stage of CKD, the kidneys permanently lose their ability to function and patients at this stage require dialysis or a kidney transplantation (KT) to survive [4].

In contrast to limited treatment options for end-stage kidney disease (ESKD), disease progression in earlier stages of CKD can be effectively delayed with appropriate management strategies. The care plan for non-dialysis-dependent CKD (NDD-CKD) patients typically includes pharmacological interventions, glycemic and blood pressure control, lifestyle modifications, and avoidance of nephrotoxic substances [5, 6]. Successful implementation of these strategies requires active patient participation and engagement in self-management practices.

Self-management plays a key role in the care of patients with chronic conditions. Clark et al. [7] defined self-management as “day-to-day tasks an individual must undertake to control or reduce the impact of disease on physical health status”. It encompasses practices such as self-monitoring, symptoms management, and other related activities. Although in the literature, the terms “self-management” and “self-care” are often used interchangeably, self-care is a broader concept applicable to all individuals, which emphasizes general health promotion and disease prevention. Self-management concentrates on managing specific health conditions and their impacts and is specific to people with chronic conditions [8].

Research has shown that better self-management of chronic diseases improves clinical outcomes, reduces healthcare costs, and improves quality of life [9]. The results of a prospective 5-year Chronic Renal Insufficiency Cohort (CRIC) Study with 3,939 participants showed that implementation of recommended CKD self-management behaviors was associated with a lower risk of disease progression, death, and atherosclerotic events [10].

For patients in the pre-dialysis stages of CKD, self-management behaviors include medication adherence, blood pressure and glucose level control, dietary modification, and physical activity. The importance of appropriate patient counseling regarding these behaviors is emphasized in the Kidney Disease: Improving Global Outcomes (KDIGO) 2024 CKD care guidelines [11]. As the levels of adherence to any recommended behavior always vary between patients, there is a need to understand the factors that influence the ability of patients with CKD to adhere to these recommended self-management practices. Several studies have investigated specific aspects of self-management behaviors and their determinants in CKD patients, but a thorough analysis of the collective evidence across studies was not available. This study therefore aims to address this gap by systematically reviewing the existing evidence on factors associated with better or poorer self-management in NDD-CKD patients. The findings of this review should help healthcare professionals and researchers to develop new or modify and improve existing self-management interventions and support strategies for patients with CKD. Better self-management in patients with CKD is expected to delay disease progression and improve quality of life.

## Methods

This systematic review followed the methodological instructions of the PRISMA guidelines (Supplementary Table S1) [12]. The protocol for this study was registered with PROSPERO (registration number CRD42024547304).

### Eligibility criteria

Studies were included in the review if they:

- Were cross-sectional, randomized controlled trials (RCTs), prospective or retrospective cohort studies, or case-control studies published from 1st January 2010 to 30th June 2024.
- Included adults (aged ≥18 years) diagnosed with CKD (as diagnosed using any recognized diagnostic criteria).
- Used a validated instrument for assessing self-management as a combination of different aspects of it.
- Provided information on the association between different factors and self-management scores.

Studies were excluded if they were conducted exclusively on patients with CKD Stage 1 and 2 or included patients below the age of 18 years, receiving dialysis, kidney transplant recipients, or patients with acute kidney injury. Review articles, case reports, editorials, conference abstracts, qualitative studies, trial protocols, non-English language studies, and studies published before 2010 were also excluded.

### Search strategy

A systematic search was conducted from July to August 2024 to identify studies that examined the association between different factors and self-management behavior. The following databases were searched: PubMed, Embase, Scopus, Medline, and CINAHL Plus. Additionally, an Internet search (Google Scholar) was conducted, and the reference lists of the included studies were screened for additional eligible studies. The search was performed using a combination of MeSH terms, free-text keywords, and Boolean operators. The language and publication date filters were applied. The full search strategy can be found in Supplementary Table S2.

### Data extraction and synthesis

The search results were managed using Zotero (Corporation for Digital Scholarship, George Mason University, Virginia, USA). Data extraction process included two stages: title and abstract screening and full-text review. Two review authors (A.M. and D.K.) independently performed data extraction. Disagreements were resolved through discussion with a third review author (A.S.S.). The data from the included studies were extracted into Excel using a standardized data extraction form. The extracted data included author, year, country, study design, sample size, population details (CKD stage, age and gender distribution, comorbidities), tools used to assess self-management, and associations of variables with self-management scores (direction and statistical significance).

Heterogeneity in reported associations was examined qualitatively by comparing included studies according to population characteristics, self-management assessment tool, determinant definitions, statistical analysis method, and quality appraisal rating. Because the included studies differed substantially in these aspects and did not report sufficiently comparable effect estimates, no common effect metric was available, no statistical assessment of heterogeneity was performed, and meta-analysis was not feasible. Therefore, findings were synthesized narratively and descriptively, with consideration of the SWiM reporting guidance for synthesis without meta-analysis [13]. The synthesis focused on the direction and statistical significance of reported associations between each determinant and self-management scores. Findings were presented in a grouped tabular form with studies grouped based on the data analysis methods utilized. Variables that were shown to be associated with self-management were combined into main categories: sociodemographic and lifestyle factors, health knowledge and literacy, clinical characteristics, biochemical/physical measurements, and psychosocial factors. In summarizing the evidence, greater interpretive weight was given to determinants examined in multiple studies, associations reported in the same direction across studies and statistically significant findings. Determinants investigated in only one study, or those with inconsistent direction or significance across studies, were interpreted more cautiously. The description of validated instruments for measuring self-management in the included studies was provided separately.

In several studies included in this review the effect of self-efficacy as one of the variables was analyzed. Self-efficacy is defined as “the belief in one’s capabilities to organize and execute the courses of action required to manage prospective situations” [14].

### Quality appraisal

Two review authors (A.M. and D.K.) independently assessed the quality of the included studies using a Newcastle-Ottawa Quality Assessment Scale adapted for the cross-sectional studies [15]. The scale included evaluating the selection, comparability, and outcome of the selected studies. Each study was categorized as very good, good, satisfactory or unsatisfactory. Discrepancies were resolved through discussion or consultation with a third reviewer (AS-S).

## Results

**FIGURE 1:**
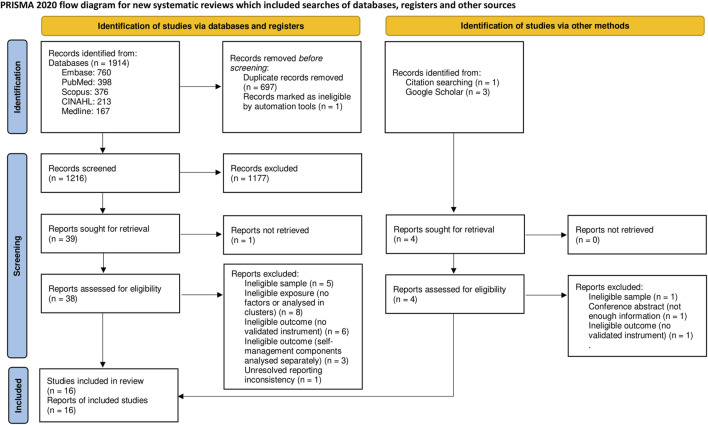
PRISMA flow diagram: study selection process (systematic review, all countries, 2010–2024).

The literature search yielded 1,914 studies. After excluding duplicated studies, 1,216 records were reviewed by title. After examining the abstracts and full text, 39 publications were selected for eligibility assessment from which 16 studies were selected to be included in this study. The detailed study selection process is illustrated in Figure 1. The quality of the studies ranged from 5 to 9 points, with six studies rated as satisfactory, six as good, and four as very good. The quality appraisal results are summarized in Supplementary Table S3.

### Characteristics of included studies

All study designs were cross-sectional. Overall, the number of participants in the included studies was 3,658. The publication years ranged from 2012 to 2023; most of the studies were conducted in Taiwan (n = 8; 50%) [16–23], then in Thailand (n = 2; 12.5%) [24, 25], and one study in each in the following countries: Saudi Arabia [26], Singapore [27], Nepal [28], the USA [29], Indonesia [30], and Myanmar [31]. The included studies only focused on adult (≥18 years), non-dialysis-dependent participants with CKD stages 1–5. One study [21] analyzed two patient groups separately: patients with CKD stage 1–5 and patients with ESKD on hemodialysis; we extracted data only for the former. A detailed description of the studies included is provided in Table 1.

**TABLE 1:** Characteristics of the Included Studies (systematic review, all countries, 2010–2024).

Authors (year)	Study type	CKD stage of participants	Country	Number of participants	Age (mean (SD), years)	Sex ratio (M:F)	Self-management assessment tool
Sritarapipat et al. [25]	Cross-sectional	Stage 3–5	Thailand	216	71.07	79:137	SMBQ
Wu et al. [21]	Cross-sectional	Early-stage CKD (analyzed separately from ESRD patients)	Taiwan	81	69.95 (12.8)	54:27	CKD-SM
Lai et al. [18]	Cross-sectional	Stage 1–3	Taiwan	112	70.16 (11.59)	69:43	CKD-SM
Almutary and Tayyib [26]	Cross-sectional	Stage 3–5 not on RRT	Saudi Arabia	203	47.3 (12.1)	102:101	CKD-SM
Suarilah and Lin [30]	Cross-sectional	Stage 1-3a	Indonesia	226	56.61 (7.48);	76:150	CKD-SM
Moktan et al. [28]	Cross-sectional	Stage 2–4	Nepal	97	45.67 (11.40);	74:23	SMBQ
Photharos et al. [24]	Cross-sectional	Stage 1–3b	Thailand	275	>70% from 51 to 65 years	165:110	CKD-SM
Wang et al. [20]	Cross-sectional	Stage 1–5 not on RRT	Taiwan	449	63.9 (13.1)	256:193	CKDSC
Wint et al. [31]	Cross-sectional	Stage 2–4	Myanmar	84	57.98 (13.61)	39:45	SMBQ
Ho et al. [27]	Cross-sectional	Stage 1–5 with hypertension not on RRT	Singapore	289	63.7 (8.7)	191:98	HTN-SCP
Chuang et al. [17]	Cross-sectional	Stage 1-3a	Taiwan	130	60.34 (11.59)	81:49	CKD-SM
Wang et al. [23]	Cross-sectional	Stage 1–5 with T2DM not on RRT	Taiwan	181	66.8 (9.7)	107:74	CKDSC
Tsai et al. [19]	Cross-sectional	Stage 1–5 not on RRT	Taiwan	454	65.8 (12.1)	254:200	CKDSC
Chen et al. [16]	Cross-sectional	Stage 3b–5 not on RRT	Taiwan	220	70.14 (11.96)	128:92	CKD-SM
Yu et al. [22]	Cross-sectional	Stages 1–5 not on RRT	Taiwan	240	63.2 (12.8)	141:99	CKDSC
Schrauben et al. [29]	Cross-sectional	Stages 1–5 not on RRT	US	401	56.7 (15.8)	213:188	SDSCAA

CKD, Chronic Kidney Disease; CKDSC, Chronic Kidney Disease Self-Care tool; CKD-SM, Chronic Kidney Disease Self-Management tool; ESRD, End-Stage Renal Disease; HTN-SC, Hypertension Self-Care Profile tool; RRT, Renal Replacement Therapy; SDSCAA, Summary of Diabetes Self-Care Activities Assessment; SMBQ, Self-Management Behavior Questionnaire; T2DM, Type 2 Diabetes Mellitus.

All studies used validated tools for self-management assessment (Table 2). Most of these tools were specifically designed for patients with CKD: CKD Self-management (CKD-SM) in seven studies [16–18, 21, 24, 26, 30] and CKD Self-Care (CKDSC) in four studies [19, 20, 22, 23]. Some studies used adapted questionnaires for patients with diabetes [29] and hypertension [27]. Three studies applied the Self-Management Behavior Questionnaire (SMBQ) [25, 28, 31].

**TABLE 2:** Validated instruments used by included studies to measure self-management in chronic kidney disease (systematic review, all countries, 2010–2024).

Tool	Description
Self-management behavior questionnaire (SMBQ)	37-item instrument with 5 dimensions: Communication with healthcare providers (8 items); partnership in care (7 items); self-care activities (11 items); self-advocacy behaviors (10 items); and medication adherence behavior (1 item).
CKD self-management (CKD-SM)	29 items with a 4-point rating scale: Self-integration (11 items), problem-solving (9 items), seeking social support (5 items), and adherence to recommended regimen (4 items)
CKD self-care (CKDSC)	16-items questionnaire with total scores and 5 subscales: medication adherence, diet control, exercise, smoking behaviors, and blood pressure monitoring
Hypertension self-care profile (HTN-SCP)	Total 60 questions with 3 subscales (each consists of 20 questions) for behavior, motivation and self-efficacy focused on medication adherence, physical activity, diet restriction, alcohol consumption, smoking, home blood pressure monitoring, weight control, regular follow-up and stress management
Summary of diabetes self-care activities assessment (SDSCAA)	9 self-care behavior measures: general “healthful” diet consumption, fruits/vegetables intake, high-fat diet intake, physical activity participation, smoking, medication adherence, nephrotoxin avoidance, blood glucose testing, and foot care

Among the included studies, 6 used correlation analysis [18, 24, 25, 28, 30, 31], 5 employed non-adjusted regression analysis [16, 17, 20, 21, 26], and 5 conducted adjusted regression analysis [19, 22, 23, 27, 29]. We combined 37 independent variables that were shown to be associated with self-management behavior into main categories: sociodemographic and lifestyle factors (n = 7), health knowledge and literacy (n = 5), clinical characteristics (n = 7), biochemical/physical measurements (n = 9), and psychosocial factors (n = 9) (Table 3).

**TABLE 3:** Reported Associations of Variables with Self-Management Behavior Score in Chronic Kidney Disease Patients (systematic review, all countries, 2010–2024).

​	​	Correlation analysis						Non-adjusted multiple regression analysis					Adjusted multiple regression analysis				
​	​	Sritarapipat et al. [25]	Lai et al. [18]	Suarilah and Lin [30]	Moktan et al. [28]	Photharos et al. [24]	Wint et al. [31]	Almutary and Tayyib [26]	Wang et al. [20]	Wu et al. [21]	Chuang et al. [17]	Chen et al. [16]	Ho et al. [27]	Wang et al. [23]	Tsai et al. [19]	Yu et al. [22]	Schrauben et al. [29]
Sociodemographic and lifestyle factors	Age	​	​	​	​	​	​	​	​	​	​	​	​	​	​	​	​
	Gender (Male)	​	​	​	​	​	​	​	​	​	​	​	​	​	​	​	​
	Marital status (married)	​	​	​	​	​	​	​	​	​	​	​	​	​	​	​	​
	Financial independence (Yes)	​	​	​	​	​	​	​	​	​	​	​	​	​	​	​	​
	Employment status (employed)	​	​	​	​	​	​	​	​	​	​	​	​	​	​	​	​
	Education level	​	​	​	​	​	​	​	​	​	​	​	​	​	​	​	​
	Smoking (Yes)	​	​	​	​	​	​	​	​	​	​	​	​	​	​	​	​
Health knowledge and literacy	Objective CKD knowledge	​	​	​	​	​	​	​	​	​	​	​	​	​	​	​	​
	Perceived CKD knowledge	​	​	​	​	​	​	​	​	​	​	​	​	​	​	​	​
	Health literacy	​	​	​	​	​	​	​	​	​	​	​	​	​	​	​	​
	Self-care knowledge	​	​	​	​	​	​	​	​	​	​	​	​	​	​	​	​
	Participation in CKD care program	​	​	​	​	​	​	​	​	​	​	​	​	​	​	​	​
Clinical characteristics	Comorbid conditions	​	​	​	​	​	​	​	​	​	​	​	​	​	​	​	​
	Hypertension	​	​	​	​	​	​	​	​	​	​	​	​	​	​	​	​
	Physical function	​	​	​	​	​	​	​	​	​	​	​	​	​	​	​	​
	Cognitive function	​	​	​	​	​	​	​	​	​	​	​	​	​	​	​	​
	CKD stage	​	​	​	​	​	​	​	​	​	​	​	​	​	​	​	​
	CKD duration	​	​	​	​	​	​	​	​	​	​	​	​	​	​	​	​
	Htn duration	​	​	​	​	​	​	​	​	​	​	​	​	​	​	​	​
Biochemical/Physical measurements	BMI	​	​	​	​	​	​	​	​	​	​	​	​	​	​	​	​
	SBP	​	​	​	​	​	​	​	​	​	​	​	​	​	​	​	​
	eGFR	​	​	​	​	​	​	​	​	​	​	​	​	​	​	​	​
	Hemoglobin	​	​	​	​	​	​	​	​	​	​	​	​	​	​	​	​
	Albumin	​	​	​	​	​	​	​	​	​	​	​	​	​	​	​	​
	Cholesterol	​	​	​	​	​	​	​	​	​	​	​	​	​	​	​	​
	Triglycerides	​	​	​	​	​	​	​	​	​	​	​	​	​	​	​	​
	LDL	​	​	​	​	​	​	​	​	​	​	​	​	​	​	​	​
	HbA1c	​	​	​	​	​	​	​	​	​	​	​	​	​	​	​	​
Psychosocial factors	Anxiety	​	​	​	​	​	​	​	​	​	​	​	​	​	​	​	​
	Depression	​	​	​	​	​	​	​	​	​	​	​	​	​	​	​	​
	Psychological wellbeing	​	​	​	​	​	​	​	​	​	​	​	​	​	​	​	​
	Self-efficacy	​	​	​	​	​	​	​	​	​	​	​	​	​	​	​	​
	Self-integration	​	​	​	​	​	​	​	​	​	​	​	​	​	​	​	​
	Problem-solving	​	​	​	​	​	​	​	​	​	​	​	​	​	​	​	​
​	Seeking social support	​	​	​	​	​	​	​	​	​	​	​	​	​	​	​	​
	Family functioning	​	​	​	​	​	​	​	​	​	​	​	​	​	​	​	​
	Social support	​	​	​	​	​	​	​	​	​	​	​	​	​	​	​	​

Each cell summarizes the association reported in the corresponding study between the listed variable and self-management score. Blue indicates a statistically significant positive association; dark red indicates a statistically significant negative association; orange indicates a non-significant association; and gray indicates that the variable was not investigated in that study. Blank cells (grey) indicate that the association was not reported. Studies are grouped according to the type of statistical analysis used: correlation analysis, unadjusted regression analysis, and adjusted regression analysis.

Study codes: A2022 = Almutary & Tayyib, 2022; C2020 = Chuang et al., 2020; C2022 = Chen et al., 2022; H2022 = Ho et al., 2022; L2021 = Lai et al., 2021; M2019 = Moktan et al., 2019; P2018 = Photharos et al., 2018; S2012 = Sritarapipat et al., 2012; S2019 = Schrauben et al., 2019; S2021 = Suarilah & Lin, 2021; T2021 = Tsai et al., 2021; W2019 = Wang et al., 2019; W2022 = Wu et al., 2022; W2023 = Wint et al., 2023; Wa2023 = Wang et al., 2023; Y2021 = Yu et al., 2021.

Abbreviations: BMI, Body Mass Index; CKD, Chronic Kidney Disease; eGFR, Estimated Glomerular Filtration Rate; HbA1c, Glycosylated Hemoglobin; Htn, hypertension; LDL, Low-density lipoprotein; SBP, Systolic Blood Pressure.

### Sociodemographic and lifestyle factors

As shown in Table 3, two studies reported that higher education levels were positively associated with self-management scores (n = 561) [18, 20], while the other two studies did not find an association between these variables (n = 384) [23, 26]. The association between age and self-management was inconsistent between studies: positive in three (n = 1,084) [19, 20, 23], negative in two (n = 423) [16, 26], and absent in two studies (n = 307) [21, 30]. Two studies (n = 635) found a significantly positive association between financial independence and self-management scores [19, 23]. One study (n = 449) has shown that married patients with CKD exhibit better self-management behaviors [20], and in one study, this association was insignificant (n = 454) [19].

### Knowledge and health literacy

Objective CKD-related knowledge demonstrated a consistent positive statistically significant association with self-management across seven studies (n = 1,365) [17, 19, 23, 25, 26, 28, 31]. A study conducted in US (n = 401) showed a significant correlation between perceived CKD knowledge and self-management [29]. Health literacy was also found to be significantly positively correlated with self-management in 5 studies (n = 1,114) [22, 24, 27, 30, 31] and lack of association between these variables was reported by one study (n = 401) [29]. A statistically significant positive association between self-care knowledge and self-management was reported by 2 studies (n = 301) [16, 21].

### Clinical characteristics

Significant associations were rarely observed between self-management and the factors included in this group. The CKD stage was positively associated with self-management score in one study (n = 449) [20]. Four studies reported the absence of a significant association between CKD duration and self-management scores (n = 956) [16, 20, 26, 31].

### Biochemical/physical measurements

In two studies (n = 652), BMI showed an inverse correlation with self-management [20, 26], whereas one study reported no such association (n = 454) [19]. Biochemical parameters were not significantly associated with self-management scores, except for HbA1c, which demonstrated a significant inverse correlation with self-management in two studies (n = 635) [19, 23].

### Psychosocial factors

Self-efficacy demonstrated a consistent significant positive association with self-management scores across seven studies (n = 946) [17, 18, 21, 25, 28, 30, 31]. Social support also showed a consistent significant positive correlation in the three studies (n = 575) [24, 25, 31]. Two studies found an inverse correlation between self-management and depression level (n = 193) [18, 21] and one study reported an inverse correlation between self-management and anxiety (n = 81) [21].

## Discussion

This systematic review summarized the evidence on factors associated with self-management among NDD-CKD patients. Our findings revealed that several sociodemographic, clinical, and psychosocial factors, either individually or in complex interplay, may influence patients’ ability to engage in effective self-management practices.

This review highlights the significant role of the levels of knowledge and health literacy in self-management behaviors of patients with CKD. Higher health literacy scores were consistently associated with better self-management scores in multiple studies. A scoping review of 28 literature reviews showed that patients with limited health literacy experience difficulties in specific domains of self-management, such as medical management, communication, and navigation of the healthcare system [32]. A recent systematic review of 48 studies investigating the role of health literacy specifically in CKD self-management also reported that higher health literacy was associated with better self-management behaviors [33]. This is supported by another scoping review investigating the link between health literacy, CKD-related knowledge, and self-management, although communicative and critical health literacy dimensions in this review were found to be more determinative than functional health literacy [34]. The observed strong influence of CKD-related knowledge can be partially attributed to its close interconnection with health literacy. A systematic review of 31 studies reported a consistent association between low health literacy and poor disease knowledge for multiple chronic diseases [35]. Van der Gaag et al. [32] have also demonstrated a correlation between CKD knowledge and health literacy. However, these factors may also have synergistic or integrated effects on self-management.

The effects of age and gender on CKD self-management were inconclusive among the included studies. These factors may not be determinative in overall self-management capacity, and their effects may rather be influenced by other factors. The observed associations between self-management and socioeconomic conditions were more consistent. Patients who were financially independent and had higher education levels had better self-management behaviors. This association was also observed among patients with type 2 diabetes [36] and chronic diseases in general [37]. Indeed, poor socioeconomic determinants can reduce a patient’s capacity for self-management, limiting his access to treatment and other resources and impacting psychological wellbeing [38]. However, as only a few studies examined the association between socioeconomic factors and self-management, more research is required to draw definitive conclusions.

Social support was positively associated with CKD self-management in the three studies included in this review [24, 25, 31]. One study also reported a positive association between being married and better self-management [20]. Marriage and social support are indeed closely linked. Marriage often provides a built-in source of emotional, practical, and social support, although the quality of relationships influences the extent of this benefit. Our results show that the presence of a supportive social network, whether through family, friends, or a spouse, may play a crucial role in managing CKD. Whitehead et al. [39] in their systematic review concluded that family support plays an important role in promoting self-management of chronic conditions. Another systematic review showed that integrating family support into self-management education for patients with type 2 diabetes improves their self-management behaviors and health outcomes [40]. A meta-analysis of 17 studies found that peer support significantly improves the self-management of patients with diabetes [41].

We also observed a consistent positive association between self-efficacy and self-management of CKD across eight studies. The results observed in the included studies align well with broader behavior theories, such as Bandura’s Social Cognitive Theory, which posits self-efficacy as a key determinant of behavior change and maintenance [14]. Self-efficacy was later incorporated into the Health Belief Model as a factor influencing health behavior. This model suggests that self-efficacy, in conjunction with other internal individual factors, explains people’s engagement, or lack thereof, in health-promoting behavior, including preventive or self-management actions [42]. In the context of CKD, higher self-efficacy likely empowers patients to take more active roles in their care, adhere to complex treatment regimens, and persist in the face of challenges.

The findings of this review have multiple implications for clinical practice and policy. In clinical practice, healthcare workers should take into account patients’ level of knowledge about CKD, health literacy, self-efficacy, and available social support when planning self-management support. Patient education should be delivered with the use of clear, accessible, and culturally appropriate communication, considering the ability of patients to understand medical information. Several educational interventions, such as those combining educational booklets, presentations, and face-to-face meetings with dietitians and social workers, have already proven their effectiveness in improving the self-management of patients and treatment outcomes [36]. Interventions strengthening self-efficacy, such as goal setting, motivational counselling, problem-solving support, and follow-up feedback, may help patients to engage more actively in self-management [43]. Considering the identified link between social support and self-management, involvement of family members, caregivers, peer-support groups, and community health workers may be useful where appropriate. At the policy level, CKD care programs should integrate structured education of patients, materials sensitive to health literacy, and psychosocial support in routine management of NDD-CKD.

The review has several limitations that should be noted. First, the meta-analysis of included studies was not possible because for many variables, insufficient studies reported associations. For more frequently studied variables like knowledge, health literacy, and self-efficacy, the methods of measurement varied significantly between studies not only in terms of tools and scales used but also in terms of operational definitions of those constructs. There was also a large heterogeneity of the included studies in terms of populations, quality, statistical methods, and tools used to assess self-management. As this review used synthesis without meta-analysis, the findings should be interpreted as patterns of association. The narrative synthesis allowed comparison of the direction and consistency of associations across studies, but it did not allow estimation of the magnitude of effects. In addition, grouping determinants into broad conceptual categories improved interpretability but may have simplified complex relationships between individual, psychosocial, clinical, and health-system factors.

Second, the predominance of cross-sectional studies in this review limits our ability to infer causal relationships. In particular, when interpreting observed associations, it is important to consider reverse causality. For example, although greater knowledge about CKD, health literacy, and self-efficacy improve self-management, patients who are already caring for themselves more actively may also seek more information, interact with healthcare workers more frequently, and, consequently, report higher levels of knowledge, health literacy, and self-efficacy. Similarly, better self-management may improve psychosocial wellbeing rather than psychosocial factors only acting as antecedents of self-management. Therefore, observed associations should be interpreted as bidirectional and interrelated.

The third limitation is the geographical concentration of studies in Asian countries, particularly Taiwan. This may limit the generalizability of the findings to other geographical and cultural contexts. The geographical concentration of studies may also have implications beyond statistical generalizability. Cultural norms and health-system characteristics can shape how patients understand and practice self-management [44]. For example, in settings where family involvement in illness management is common, social support may play a particularly important role in medication adherence, dietary modification, communication with healthcare providers, and monitoring of symptoms [39]. Similarly, patient-provider communication patterns, access to nephrology or primary care services, and affordability of medications or laboratory monitoring may influence patients’ ability to engage in self-management [37]. Therefore, determinants identified in this review may not operate in the same way across countries with different cultural expectations, healthcare financing models, and organization of CKD care. Future research should include more populations from other regions of the world.

Another limitation is that many studies primarily focused on measuring the effect of frequently reported variables such as knowledge, health literacy, and self-efficacy. While these variables showed strong positive associations, the exploration of other factors was comparatively less extensive. This suggests a potential research bias towards well-established determinants, potentially overlooking other emerging or less-studied factors that might also significantly influence self-management behaviors in CKD patients. Despite these limitations, strengths of this study include the rigorous methodology and quality assessment of included studies that ensure robustness of our findings. Hence, factors identified to be associated with self-management highlight areas of focus for improving existing care strategies for people with CKD.

## Conclusion

This systematic review provides comprehensive evidence on factors associated with self-management in NDD-CKD patients. The findings highlight the crucial role of modifiable factors, particularly disease-related knowledge, health literacy, and self-efficacy, in promoting effective self-management behaviors. Healthcare providers should focus on developing and implementing interventions that enhance these aspects while considering patients’ social support systems. Future research should employ longitudinal designs to establish causal relationships and include more diverse geographical populations.

## Data Availability

The original contributions presented in this study are included in the article/Supplementary Material, further inquiries can be directed to the corresponding authors.
