# Supplementary material for: Key determinants of self-management in patients with non-dialysis-dependent chronic kidney disease: a systematic review
Source: Public Health Rev. 2026 Jun 30;47:1609108. doi: 10.3389/phrs.2026.1609108 (PMC13364728; doi:10.3389/phrs.2026.1609108)
Supplement: Supplementary file 2 [file Table2.DOCX]

**Table S2.** Search Strategy for Identifying Studies on Factors Associated with Self-Management in Chronic Kidney Disease

| Database | Keywords |
| --- | --- |
| Medline | 1. ‘chronic kidney disease’ OR ‘chronic renal disease’ OR ‘chronic renal failure’ OR ‘chronic kidney failure’ OR ‘ckd’ OR ‘chronic renal insufficiency’ OR ‘chronic kidney insufficiency’ 2. ‘self-management’ OR ‘self management’ OR ‘self-care’ OR ‘self care’ 3. Adults 19+ (filter) 4. English language (filter) 5. Full text (filter) 6. 1 AND 2 AND 3 AND 4 AND 5 |
| Pubmed | ((((((("chronic kidney disease") OR ("chronic renal insufficiency")) OR ("chronic renal disease")) OR ("chronic kidney insufficiency")) OR ("chronic kidney failure")) OR ("chronic renal failure")) OR (CKD) AND ((fft[Filter]) AND (english[Filter]) AND (alladult[Filter]) AND (2010:2024[pdat]))) AND (((("self-management") OR ("self management")) OR ("self-care")) OR ("self care") AND ((fft[Filter]) AND (english[Filter]) AND (alladult[Filter]) AND (2010:2024[pdat]))) |
| CINAHL | 1. ‘chronic kidney disease’ OR ‘chronic renal disease’ OR ‘chronic renal failure’ OR ‘chronic kidney failure’ OR ‘ckd’ OR ‘chronic renal insufficiency’ OR ‘chronic kidney insufficiency’ 2. ‘self-management’ OR ‘self management’ OR ‘self-care’ OR ‘self care’ 3. Adults 19+ (filter) 4. English language (filter) 5. Full text (filter) 6. 1 AND 2 AND 3 AND 4 AND 5 |
| EMBASE | 1. ('chronic kidney disease' OR 'chronic renal insufficiency' OR 'chronic renal disease' OR 'chronic kidney insufficiency' OR 'chronic kidney failure' OR 'chronic renal failure' OR ckd) AND [english]/lim AND ([adult]/lim OR [aged]/lim) AND [2010-2024]/py 2. ('self-management' OR 'self management' OR 'self-care' OR 'self care') AND [english]/lim AND ([adult]/lim OR [aged]/lim) AND [2010-2024]/py 3. #1 AND #2 |
| Scopus | ( &apos;chronic AND kidney AND disease&apos; OR &apos;chronic AND renal AND disease&apos; OR ckd OR &apos;chronic AND renal AND insufficiency&apos; OR &apos;chronic AND kidney AND insufficiency&apos; OR &apos;chronic AND renal AND failure&apos; OR &apos;chronic AND kidney AND failure&apos; ) AND ( &apos;self AND management&apos; OR &apos;self-management&apos; OR &apos;self AND care&apos; OR &apos;self-care&apos; ) AND ( adult OR adults ) AND ( LIMIT-TO ( LANGUAGE , "English" ) ) |
