# Supplementary material for: Key determinants of self-management in patients with non-dialysis-dependent chronic kidney disease: a systematic review
Source: Public Health Rev. 2026 Jun 30;47:1609108. doi: 10.3389/phrs.2026.1609108 (PMC13364728; doi:10.3389/phrs.2026.1609108)
Supplement: Supplementary file 3 [file Table3.DOCX]

**Table S3.** Quality Assessment of Cross-sectional Studies Using Modified Newcastle-Ottawa Scale

|  | Item & score | | | | | | | | |
| --- | --- | --- | --- | --- | --- | --- | --- | --- | --- |
|  | Selection | | | | Comparability | Outcome | |  |  |
| Study | Representativeness of the sample | Sample size | Non-Response rate | Ascertainment of the exposure (risk factor) | Comparability of subjects in different outcome groups on the basis of design or analysis. Confounding factors controlled | Assessment of outcome | Statistical test | Total | Interpretation |
| Sritarapipat et al., 2012 | - | * | * | ** | - | ** | - | 6 | Satisfactory |
| Wu et al., 2022 | - | * | * | ** | ** | ** | * | 9 | Very Good |
| Lai et al., 2021 | - | - | - | ** | ** | ** | * | 7 | Good |
| Almutary et al., 2022 | - | * | * | ** | ** | ** | * | 9 | Very Good |
| Suarilah et al., 2022 | - | - | * | ** | - | ** | * | 6 | Satisfactory |
| Moktan et al., 2019 | - | * | * | ** | - | ** | - | 6 | Satisfactory |
| Photharos et al., 2018 | - | - | * | ** | - | ** | - | 5 | Satisfactory |
| Wang et al., 2023 | - | - | * | ** | ** | ** | * | 8 | Good |
| Wint et al., 2023 | - | * | * | ** | ** | ** | * | 9 | Very Good |
| Ho et al., 2022 | * | * | * | ** | ** | ** | - | 9 | Very Good |
| Chuang et al., 2021 | - | * | * | ** | ** | ** | - | 8 | Good |
| Wang et al., 2019 | - | - | - | * | ** | ** | * | 6 | Satisfactory |
| Tsai et al., 2021 | - | - | * | * | ** | ** | * | 7 | Good |
| Chen et al., 2022 | - | * | * | * | ** | ** | - | 7 | Good |
| Yu et al., 2021 | - | - | * | * | ** | ** | * | 7 | Good |
| Schrauben et al., 2018 | - | - | - | * | ** | ** | * | 6 | Satisfactory |

*Note:* * = 1 point; ** = 2 points; - = 0 points. Maximum possible score is 10 points. Quality rating interpretation: Very Good (9-10 points), Good (7-8 points), Satisfactory (5-6 points).
